# A Pilot Case Series on the Use of a Large Mushroom-Shaped Corneal Graft for the Surgical Management of Post-Penetrating Keratoplasty Ectasia and Endothelial Failure

**DOI:** 10.3390/jcm14020343

**Published:** 2025-01-08

**Authors:** Antonio Moramarco, Luigi Fontana, Natalie di Geronimo, Giulio Rapezzi, Giacomo Savini, Pietro Viola, Maurizio Mete, Vito Romano

**Affiliations:** 1 IRCCS Azienda Ospedaliero-Universitaria di Bologna, 40126 Bologna, Italy; moramarco.antonio@libero.it (A.M.); natalie.digeronimo@outlook.it (N.d.G.); giulio.rapezzi@gmail.com (G.R.); mau.mete@gmail.com (M.M.); 2 Ophthalmology Unit, Dipartimento di Scienze Mediche e Chirurgiche, Alma Mater Studiorum University of Bologna, Via Palagi 9, 40138 Bologna, Italy; 3 IRCCS Bietti Foundation, 00198 Rome, Italy; giacomo.savini@startmail.com; 4 Ophthalmology Unit, San Bortolo Hospital, 36100 Vicenza, Italy; dr.pietro.viola@gmail.com; 5 Eye Unit, Department of Medical and Surgical Specialties, Radiological Sciences, and Public Health, University of Brescia, 25123 Brescia, Italy; vito.romano@gmail.com

**Keywords:** corneal endothelium, corneal transplantation, eye bank, tissue donor, penetrating keratoplasty, corneal ectasia, keratoconus

## Abstract

**Objective**: The aim of this study was to evaluate the effect of a surgical technique for managing post-penetrating keratoplasty (PK) ectasia complicated by late endothelial failure (LEF). **Methods**: A single-center pilot case series was conducted regarding consecutive patients affected by post-PK ectasia with late graft failure. Using a microkeratome, a single donor cornea was dissected to prepare a two-piece graft, comprising a larger anterior lamella made up of anterior stroma and a smaller posterior lamella made up of posterior stroma, Descemet’s membrane, and endothelium. The two lamellae were then positioned on the appropriately prepared recipient cornea. The technique was applied to 15 patients between 2022 and 2023, and data were retrospectively collected from preoperative evaluations and at 1, 6, and 12 months, post-operatively. At each visit, patients underwent standard clinical evaluation, corneal topography, and endothelial cell density evaluation, and visual acuity was measured using a LogMAR chart. **Results**: The technique restored normal corneal curvature and achieved a clear graft in all patients, leading to the resolution of preoperative ectasia and improved corneal pachymetry. At the one-year follow-up, the average K was reduced from 51.1 ± 4.5 D to 43.5 ± 1.1 D; the best corrected visual acuity (BCVA) was improved from 1.1 ± 0.4 to 0.3 ± 0.2 LogMAR; the central corneal thickness was reduced from 629 ± 39 μm to 532 ± 45 µm; and the endothelial cell density was 1926 ± 199 cells/mm^2^. None of the patients developed severe complications. **Conclusions**: The two-piece manual mushroom PK may represent an effective technique for managing complex post-PK ectasia cases combined with endothelial decompensation.

## 1. Introduction

**Figure 1:**
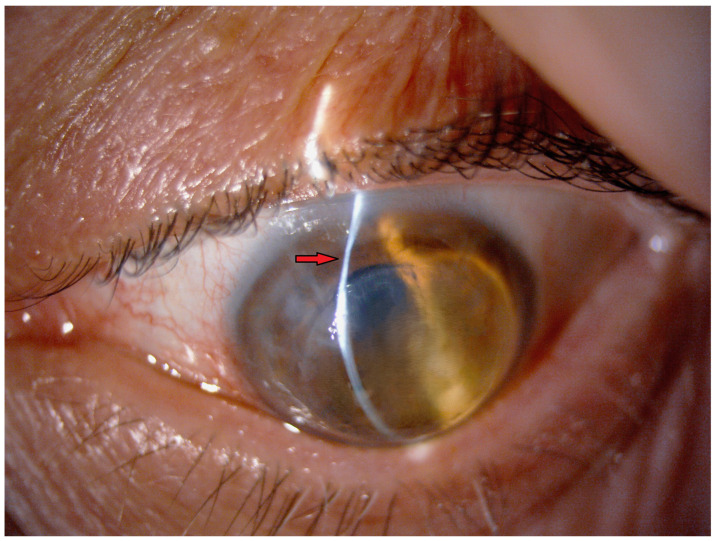
Post-PK ectasia with endothelial dysfunction. Slit-lamp examination highlights thinning of the host–donor rim (red arrow). All images of the patient’s eyes were obtained with the patient’s consent.

Ectasia after penetrating keratoplasty (PK) is a late complication that can occur after the procedure, characterized by the thinning and steepening of the cornea at the donor–recipient junction [1] (Figure 1), potentially leading to high irregular astigmatism and significant visual impairment. It can occur years or decades after PK is performed for any indication, but it is relatively rare in non-keratoconus eyes [2,3,4,5]. The exact cause of this condition is still being determined, and while several theories have been proposed, there is no definitive consensus. These theories include the thinning of the donor tissue over time, irregular suturing or abnormal healing, chronic eye rubbing, elevated intraocular pressure (IOP), and the progression of underlying keratoconus in the peripheral recipient tissue [6,7,8,9,10,11,12].

Late endothelial failure (LEF) is another potential complication of PK, defined as a gradual decline in graft transparency without a recent history of rejection and unresponsiveness to corticosteroids. This is a concern at any point after PK but becomes more common as the graft ages [13,14,15]. There are various reasons for graft failure, with non-immune-mediated endothelial cell loss (ECL) being the second most common cause after immune-mediated rejection and the most common after the first five years post-surgery [15,16,17].

The surgical management of post-PK ectasia is typically considered a last resort after exploring less invasive options like scleral contact lens fitting, corneal cross-linking (CXL), or photorefractive keratectomy. Transplant surgery is warranted when these methods prove ineffective or become intolerable for the patient [18]. However, surgical options are limited when the eye presents both corneal ectasia and graft endothelial decompensation, as techniques that aim to spare the existing endothelium are unsuccessful in these cases.

This study reports the surgical outcomes of a two-piece PK technique (“mushroom-shaped PK”) as a possible surgical solution for post-PK ectasia and endothelial failure.

## 2. Materials and Methods

Patients with secondary ectasia following a previous penetrating keratoplasty combined with an endothelial decompensation of the graft were selected for the surgical procedure. A combination of clinical characteristics and keratometric measurements were used to diagnose corneal ectasia. The clinical features included irregular curvature on slit-lamp examination and thinning at the graft–host junction [1]. There is no consensus in the literature on keratometric values for diagnosing post-PK ectasia. Still, based on similar patient populations in other studies, we considered either an average keratometry power (AvK) ≧ 48 D or the presence of irregular astigmatism ≧ 8 D to be indicative of ectasia [3,5]. Endothelial dysfunction was defined as a central corneal thickness CCT > 600 µm or evidence of corneal edema without signs of rejection under slit-lamp examination performed by two expert examinators (L.F. and A.M.).

The exclusion criteria included a history of ocular surgery excluding primary PK; significant ocular comorbidities such as glaucoma, retinal disease, or advanced cataract; and active endothelial rejection at preoperative evaluation.

After surgery, patients were followed-up at our clinic at 1 day; 1 week; and 1, 6, and 12 months post-operatively. The best corrected visual acuity (BCVA) (LogMAR), slit-lamp examination, IOP (Goldman tonometer, Haag-Streit, Koeniz, Switzerland), specular microscopy (Tomey, Nagoya, Japan), corneal topography (Oculus Optikgeraete GmbH; Wetzlar, Germany), and anterior segment optical coherence tomography (AS-OCT) (Casia II, Tomey Corp., Nagoya, Japan) results were recorded at each preoperative and post-operative visit. The number and severity of intraoperative and post-operative complications were noted. All the sutures were removed within a year of surgery, and data were collected at 1 year after the removal of sutures in all patients. The standard post-operative therapy protocol included the administration of Chloramphenicol 0.2%/Betamethasone phosphate 0.5% eyedrops, administered four times daily during the first post-operative month and then twice daily during the second and third months. After three months, these were replaced with Hydrocortisone 0.3% eye drops administered twice daily for the follow-up period.

The clinical variables BCVA and CCT are expressed using the mean ± SD. The variables were compared using a paired sample *t*-test. Statistical significance was set at *p* < 0.05. Statistical analyses were performed using SPSS for Windows version 25 (IBM Corp., Armonk, NY, USA).

## 3. Technique

### 3.1. Preoperative Planning

**Figure 2:**
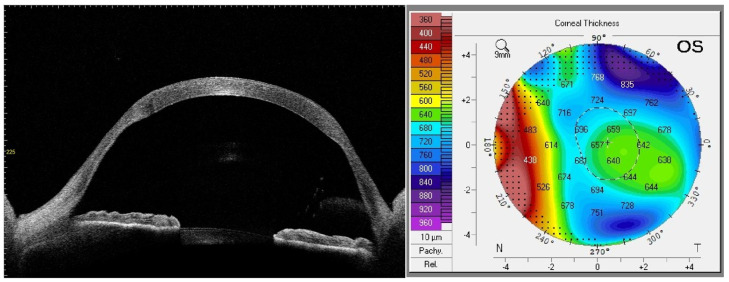
Same eye as in Figure 1. AS-OCT shows ectatic changes and thinning of the host–donor interface. Pachymetry map shows increased thickness due to corneal edema.

Anterior segment optical coherence tomography (AS-OCT) is the key imaging technique used in presurgical planning for patients with post-PK ectasia. Crucial measurements include the vertical and horizontal corneal diameters, peripheral host ring thickness, and previous PK graft diameter (Figure 2). The vertical corneal diameter, typically 11 to 12 mm, provides essential information about the cornea’s size. Host ring thickness measurements, usually ranging between 300 and 600 microns, help to determine the corneal tissue’s structural integrity near the transplantation site. AS-OCT also allows for an assessment of the previous PK graft size, typically between 7.5 and 8.5 mm in diameter. These measurements, obtained through AS-OCT, aid in selecting the appropriate graft sizes and surgical techniques tailored to the individual patient’s anatomical characteristics to achieve the best possible visual outcome and reduce the risk of rejection.

### 3.2. Donor Graft Preparation

Following the procedure described by Busin et al. [19], the donor cornea is mounted onto an Artificial Anterior Chamber (AAC) device (Moria SA, Antony, France). The donor cornea’s thickness is measured using a handheld pachymeter (Pachmate^®^, DGI I Technology Inc., Exton, PA, USA) or with intraoperative OCT (see the dedicated section). Then, the donor cornea is split into two lamellae using a microkeratome (Evolution3e^®^, Moria SA) with a head cutting depth selected based on the pachymetry of the donor cornea to obtain a posterior lamella approximately 250 microns thick. After splitting, the anterior and posterior lamellae are punched to the appropriate size. In our technique, the punch diameter of the lamellae remained consistent among cases. The anterior (larger) lamella was cut using a 9.0 mm or 9.5 mm donor punch, and the posterior lamella was punched according to the previous PK graft size, usually measuring between 7.5 mm and 8.5 mm.

### 3.3. Recipient Bed Preparation

**Figure 3:**
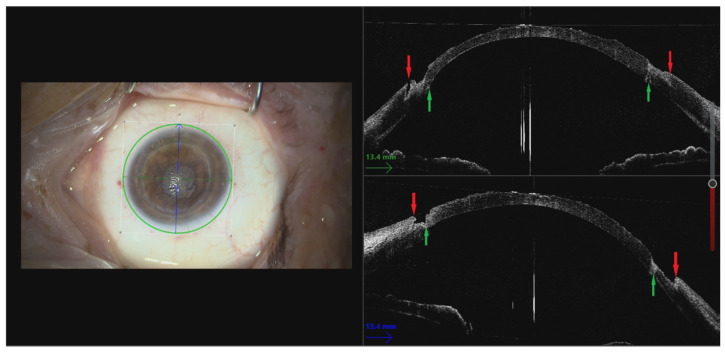
Manually dissected stromal bed on the recipient cornea. On the OCT image, the edge of the trephination (red arrows) and the donor–host junction of the previous PK (green arrows) are visible.

The recipient cornea’s thickness is measured with a handheld pachymeter or iOCT. A partial-thickness trephination, sized at 9.0 mm or 9.5 mm in diameter, is carried out using a single-use guarded corneal trephine (Moria SA), chosen according to the white-to-white measurement. The depth of the trephination is based on the recipient’s pachymetry and aims to maintain a stromal bed with a minimum thickness of 250 microns (Figure 3), prioritizing the maintenance of corneal integrity while facilitating optimal graft placement. A meticulous manual lamellar dissection is then performed across the surface of the trephination to obtain an even-thickness stromal bed.

**Figure 4:**
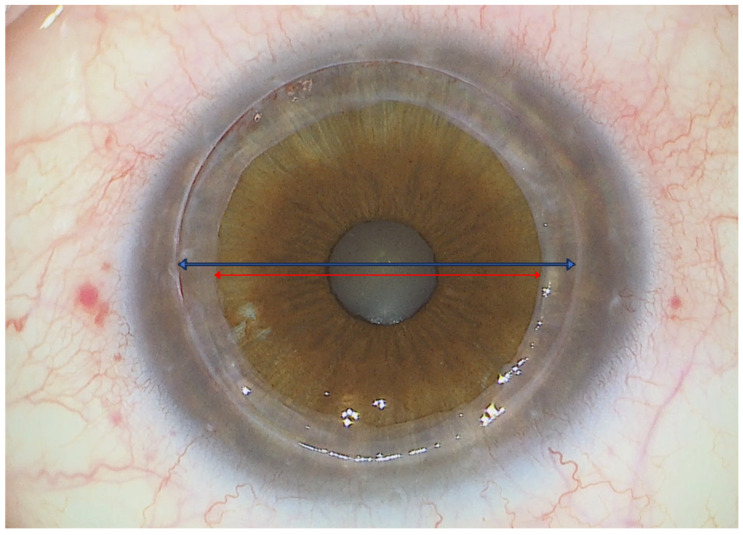
Fully prepared recipient cornea. The edge of the partial-thickness trephination (blue arrow, 9.5 mm in diameter), the peripheral stromal bed ring, and the central full-thickness opening (red arrow, 8 mm in diameter) in place of the previous PK graft are clearly visible.

Subsequently, the remaining portion of the previous corneal graft is manually excised using corneal scissors to create a full-thickness central hole measuring between 7.5 mm and 8.5 mm in diameter, depending on the size of the previous graft. This creates the scaffold to house the anterior and posterior lamella of the mushroom-shaped penetrating keratoplasty (PK) (Figure 4).

### 3.4. Graft Positioning and End of the Procedure

**Figure 5:**
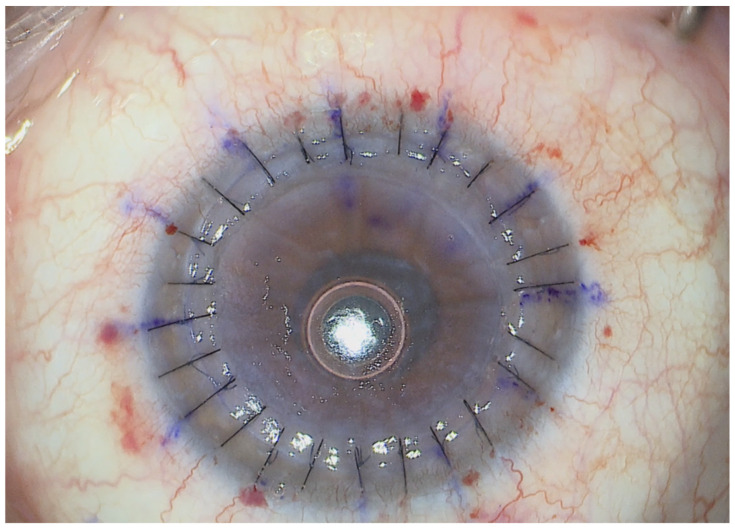
Anterior lamella sutured to the recipient cornea with 16 interrupted 10–0 nylon sutures. The posterior lamella was fitted without sutures.

The posterior lamella is placed first and fitted into the full-thickness hole left by the excised PK graft without suturing. Then, the anterior lamella is placed on top of the previous lamella, aligning its larger diameter with the recipient’s partial-thickness stromal bed. The anterior lamella is then secured with four cardinal sutures, followed by a firm attachment to the recipient cornea using either a 10–0 nylon continuous suture or 16 single sutures (Figure 5). Interrupted sutures were preferred in patients with limbal neovascularization.

In the case of insufficient adhesion between the anterior and posterior lamella at this stage, an air bubble can be injected into the AC to promote contact between the two lamellae and may be removed at the end of the procedure. However, in the case of incomplete adhesion, an inferior iridectomy is performed with a vitrectome inserted through a limbal paracentesis, and the AC is filled with air. Following our standard practice for endothelial grafts, when the AC is filled with air, the patient should be examined 2 h after surgery to check the air bubble level. If the air bubble covers the inferior iridectomy, air is partially released at the slit lamp by gently pressing on the paracentesis with a 25 G needle. In this case, the patient is instructed to maintain a face-up position for a few days post-operatively (Supplementary Materials, Video S1).

### 3.5. Use of Intraoperative OCT

**Figure 6:**
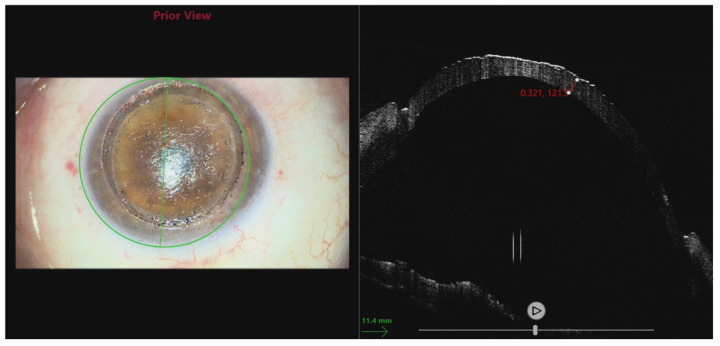
Measuring the thickness of the stromal bed using an intraoperative OCT scan.

**Figure 7:**
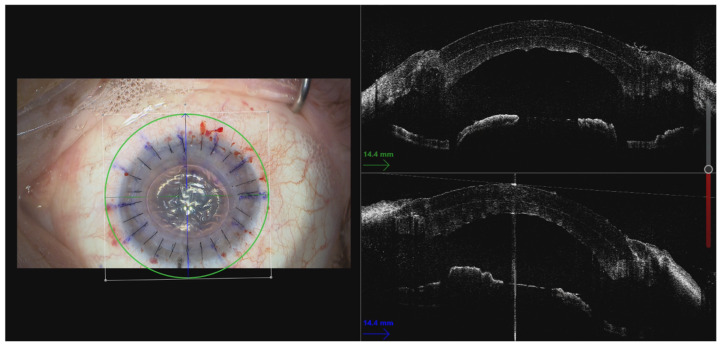
Intraoperative OCT scan at the very end of the procedure. Both the anterior and posterior lamellae can be seen. The scans show correct positioning and perfect adhesion between the lamellae, which can be easily verified by the surgeon in the operating room before closing the case.

The technique described in this paper is designed to be performed effectively and repeatably by surgeons using a standard surgical ophthalmic microscope. However, an intraoperative OCT apparatus (Enfocus OCT^®^ on the Proveo8^®^ microscope, Leica Camera AG, Wetzlar, Germany), along with a classic coaxial microscopic view, can facilitate several steps of the procedure. Intraoperative OCT imaging is an innovative technology that offers significant aid in both anterior and posterior segment surgery [20] and has become part of standard surgical practice at our center. For instance, in the procedure described, OCT can be used to intraoperatively assess the peripheral thickness of the recipient cornea and guide the initial trephination. After lamellar dissection of the recipient cornea, a real-time measurement of the thickness of the stromal bed helps in choosing the appropriate microkeratome head to match the removed tissue (Figure 6). Finally, near the end of the procedure, OCT imaging allows the surgeon to accurately verify the correct positioning and adhesion of the lamellae (Figure 7).

## 4. Results

The above-mentioned technique was applied for the treatment of 15 consecutive patients with post-PK ectasia and graft decompensation between 2022 and 2023. The patients’ mean age was 58 ± 9 years, fourteen patients were male (93.3%), and one was female, and the operated eyes included seven right eyes and eight left eyes. All patients presented significant ectasia combined with endothelial decompensation developed years after PK. The most common indication for primary PK was keratoconus (13/15, 86.6%); one patient underwent PK for a leukoma following trauma, and another had an optical PK after non-specified infectious keratitis. The year of primary surgery was not available in three patients; among the others, the most recent PK was performed 14 years beforehand in a patient with ocular trauma, while the oldest was performed 53 years beforehand for keratoconus. The number of years after primary PK was 28.5 ± 13.3. Preoperatively, AvK was 51.1 ± 4.5 D, Kmax was 56.5 ± 5.1 D, Kmin was 46.8 ± 4.9 D, astigmatism was 9.7 ± 4.6 D, BCVA was 1.1 ± 0.4 LogMAR, and the corneal thickness was 629 ± 39 μm. The preoperative endothelial cell density was not measurable in all patients due to graft opacification and the poor condition of the corneal endothelium (Table 1).

**Figure 8:**
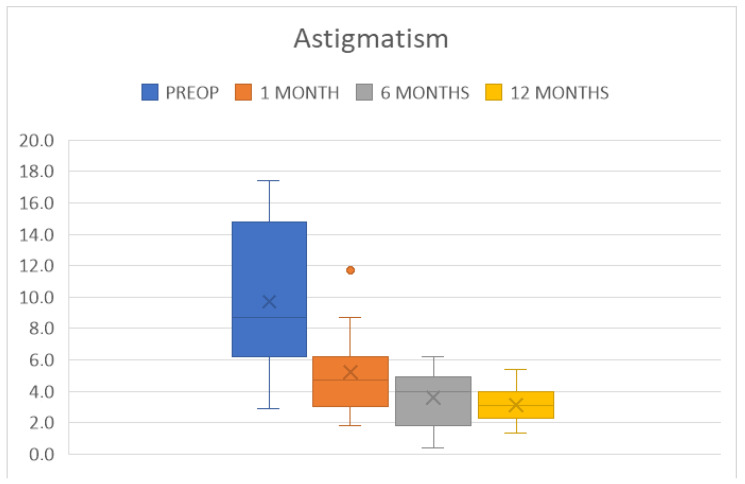
Box plot distribution of astigmatism at the chosen time points.

The average K was 43.5 ± 1.4 D at 6 months and 43.5 ± 1.1 D (*p* < 0.01) at 12 months; the average Kmin and Kmax were 41.7 ± 1.8 D and 45.3 ± 1.5 D at 6 months and 42.0 ± 1.2 D and 45.1 ± 1.2 D at 12 months (*p* < 0.01), respectively; and the residual astigmatism was 3.6 ± 1.7 D and 3.0 ± 1.1 D at 6 and 12 months, respectively (*p* < 0.01) (Figure 8). All patients had all sutures removed within a year of surgery. None of them experienced an increase in astigmatism or its irregularity following suture removal. The topometric parameters and BCVA at the 12 month examination were measured after the removal of sutures. The reduction in astigmatism before the 12 month threshold is partly attributable to the healing of the surgical wound and the regularization of the host–donor interface, which occurs during the first year post-transplant [21], and partly to the selective removal of sutures guided by topographic analysis [22].

**Figure 9:**
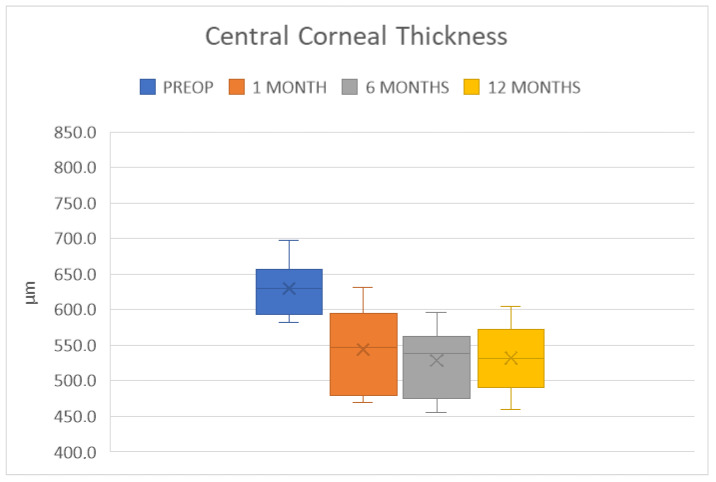
Box plot distribution of central corneal thickness at the chosen time points.

Endothelial decompensation was also addressed by the procedure, with significant improvements in the pachymetry values. The average corneal thickness at the pupil center was 544 ± 54.6 μm at 1 month, 528.4 ± 46.1 μm at 6 months, and 532.1 ± 44.6 μm at 12 months. These changes were statistically significant compared to the preoperative values (*p* < 0.001 at all time points) and did not show significant differences between 1 and 12 months post-operation (*p* = 0.12), indicating that the endothelial deturgescent function remained stable throughout the follow-up period (Figure 9. The ECD was 2527 ± 208 cells/mm^2^ at 1 month post-operatively, 2181 ± 190 cells/mm^2^ at 6 months, and 1926 ± 199 cells/mm^2^ at 12 months. The progressive loss of endothelial cells is a known phenomenon in corneal grafts, and the rate of ECL in our case series is comparable to the results reported in the previous literature [23,24,25].

**Figure 10:**
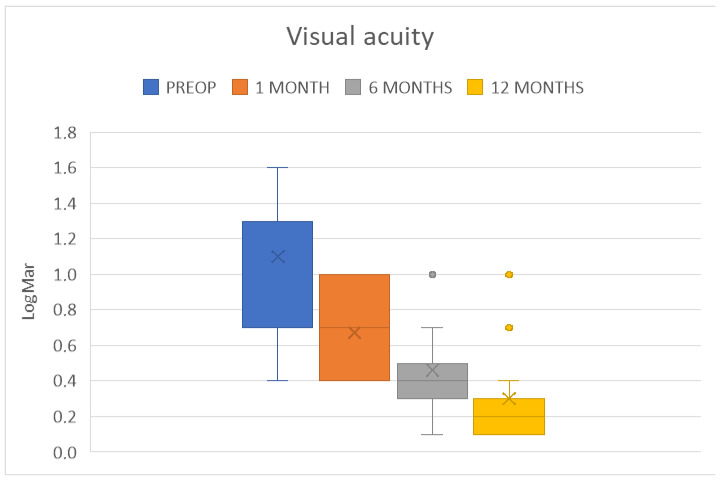
Box plot distribution of best corrected visual acuity (BCVA) at the chosen time points.

All patients had a remarkable improvement in visual acuity. The average BCVA was 0.5 ± 0.3 LogMAR at 6 months (*p* < 0.001) and 0.3 ± 0.2 LogMAR at 12 months (*p* < 0.001). Two patients (13.3%) had a final BCVA below 0.4 LogMAR (Figure 10). The results at 6 and 12 months are summarized in Table 2.

No patient had adverse events of severe entity or with a measurable impact on outcomes. One patient had an episode of mild stromal rejection at 8 months, which was managed with topical corticosteroids and rapidly resolved without sequelae.

## 5. Discussion

Post-PK ectasia is a visually debilitating condition affecting long-lasting PK grafts. Its incidence may be underestimated due to inconsistency in its definition throughout the existing literature, with terms like “recurrence of keratoconus”, “late ectasia after PK”, “ectasia of the host corneal rim”, “recurrent ectasia in corneal grafts”, and “graft protrusion” [2,9,10,26]. This complicates communication and data comparison across different studies and clinical settings. The reported incidence seems to be about 40% at 20 years after penetrating keratoplasty for keratoconus, while it is less frequent in eyes undergoing PK for other indications [1,2,3].

In post-PK ectasia, conservative management with rigid gas-permeable (RGP) contact or scleral lenses to regularize the corneal surface remains the first option [1,18]. CXL, aiming to reinforce the corneal fibers and halt or slow down the ectasia progression [27], and Topography-Guided Photorefractive Keratectomy, to normalize the irregular corneal surface [28], stand out as less invasive surgical approaches and represent adequate options in many mild-to-moderate cases. However, surgical management becomes necessary when contact lenses are no longer tolerated or fail to provide satisfactory visual acuity. Lamellar techniques such as Peripheral Anterior Lamellar Keratoplasty [26] and “Overlay” Deep Anterior Lamellar Keratoplasty (L-DALK) [29,30] have been described. L-DALK, in particular, represents a viable and effective alternative to PK for the correction of post-keratoplasty ectasia, offering all the advantages of an anterior lamellar surgery procedure and closed-eye surgery [31,32,33].

The long-term outcomes of corneal transplantation are also influenced by graft failure, which is related to progressive post-operative ECL and immunologic rejection and becomes more prevalent as the graft ages [14,16,34]. In normal eyes, 0.6% of the endothelial cells are lost yearly [35]. After penetrating keratoplasty, 45% of endothelial cells are lost in the first 3 years post-operatively and progressive ECL continues even 20 years after surgery [36]. The survival of PK grafts is reported to be 45–55% at 15 years and 44% at 20 years [13,15,16]. Primary diagnosis influences graft survival; patients with keratoconus showed the best 10-year survival estimate (95%), followed by endothelial and stromal dystrophies (both 55%), infectious leukomas (49%), trauma (33%), and chemical burns (14%) [37,38]. Redo PK, Descemet stripping automated endothelial keratoplasty (DSAEK-on-PK), and Descemet membrane endothelial keratoplasty (DMEK-on-PK) are all viable treatment options for the endothelial failure of PK. The advantages of EK-on-PK include the preservation of the previous astigmatic rehabilitation of the primary PK, faster visual rehabilitation than redo PK, and the avoidance of the use of sutures; it may be a better option in the setting of ocular surface disease. However, redo PK is the most suitable option for combined endothelial failure with stromal opacity or where the primary PK caused intolerable astigmatism before failure [39,40].

This is the case of combined ectasia post-PK and LEF, in which L-DALK and EK-on-PK would only partially resolve the problem. Around 30–35 years ago, penetrating keratoplasty became more widely utilized as a therapeutic option [41], and it represented the majority of corneal transplantations until the late 2000s [42,43]. As such, we may have already entered the time window in which the burden of post-PK ectasia is becoming more prevalent and may coexist with LEF in many cases.

A repeat penetrating keratoplasty can be considered in these patients, but such a procedure comes with a higher risk of rejection and graft failure and a poorer visual prognosis [37,44,45]. According to the Dutch registry study [46], the rate of immunologic rejection in repeated penetrating keratoplasty (PK) represents a significant concern in corneal transplantation outcomes. Irreversible rejection accounted for 20% of regraft failures in repeated PK and 17% in repeated endothelial keratoplasty (EK), highlighting the prominence of rejection as a determinant of graft survival. The overall five-year survival rate for repeated PK was 55%, markedly lower than the 81% observed for repeated EK. These data underscore the heightened immunologic challenges associated with repeated PK, particularly in cases requiring full-thickness grafts. Chronic ECL, often attributed to undetected or subtle rejection episodes, further exacerbates these outcomes and reinforces the need for robust long-term immunosuppressive regimens. Additionally, the thinning of the peripheral recipient cornea in post-PK ectasia requires the use of larger-diameter corneal grafts (8.5–9.5 mm), which have been associated with lower graft survival due to a heightened risk of graft rejection and late-stage endothelial failure, cataract formation, and an increased likelihood of post-operative glaucoma [1,3,10,16,47]. In addition, it may increase the likelihood of thickness disparity between the new graft and the recipient, leading to significant post-operative astigmatism. These considerations highlight the delicate balance required in surgical planning for repeated PK, aiming to optimize graft stability and survival while mitigating the inherent risks of increased diameter and rejection.

Another option is a two-step surgical approach, where an overlay DALK is performed first to address ectatic changes, followed by an endothelial keratoplasty to treat graft decompensation. However, this approach is less feasible in a real-life setting, as it exposes the patient to the risks associated with two separate surgical procedures, resulting in a greater expense of time and resources [48].

The microkeratome-assisted two-piece mushroom penetrating keratoplasty (“mushroom PK”) technique was pioneered by Busin et al. [19]. This technique offers the refractive advantages of a large anterior lamellar keratoplasty combined with a limited removal of the central recipient endothelium. This approach is proven to have a reduced rejection rate and prolonged survival, even in high-risk patients, such as those with post-traumatic corneal scars and vascularized corneas, due to the minimal replacement of healthy endothelium [49,50,51].

A mushroom-shaped PK can also be performed with the help of femtosecond-laser (FSL) devices to cut the donor and the recipient cornea into the desired shape [52]. Various studies have evaluated the use of single-piece femtosecond-laser-cut mushroom PK in patients with keratoconus, reporting good visual outcomes [53,54,55]. Notably, Levinger et al. also reported lower astigmatism and lower ECL compared to manual PK [54]. The laser-cut mushroom-shaped wound is more mechanically stable than a manual PK wound [56,57]. However, FSL achieves maximum precision only when operating on relatively straightforward tissue; the laser cutting of an opaque or edematous cornea can result in incomplete or irregular dissection, mainly when operating at a deeper layer, such as in penetrating keratoplasty [58,59]. The patients targeted by our technique presented with endothelial decompensation, resulting in various grades of corneal edema; therefore, FSL cutting may not achieve sufficient precision in these cases. Femtosecond-laser devices also suffer from a cost-effectiveness problem, which has been extensively documented and limits availability in a standard clinical setting [60,61,62,63].

**Figure 11:**
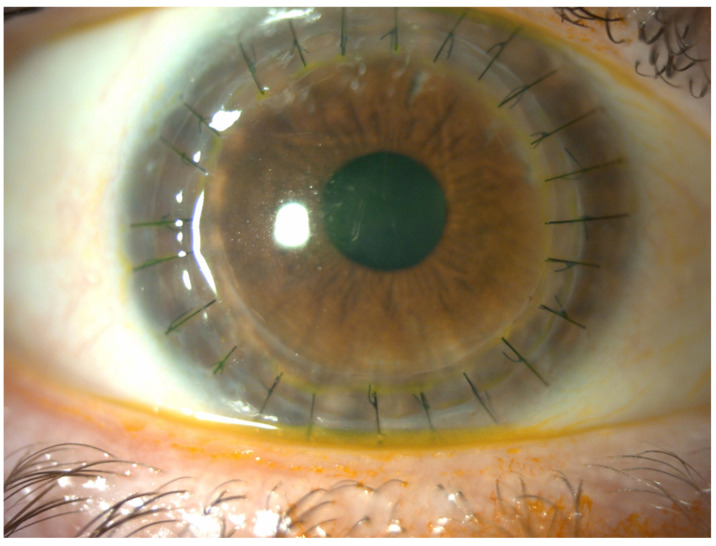
Same eye as in Figure 3, Figure 4, Figure 5, Figure 6 and Figure 7, one month post-surgery. The patient reported a significant improvement in visual acuity. The lamellae are correctly positioned, and the cornea is transparent. Sutures will be removed 12 months post-operatively.

Our technique is a manual two-piece mushroom-shaped PK, based on the technique introduced by Busin, applied to the need to correct the elevated astigmatism typical of post-PK ectasia and graft decompensation. Based on preoperative studies conducted using AS-OCT, our technique utilizes an anterior corneal lamella with the largest possible diameter. Creating the largest possible lamella helps to minimize the post-operative astigmatism values. It is well known, even in DALK, that larger diameters are associated with lower astigmatism values without increasing the risk of rejection or graft failure [64]. However, replacing an endothelial lamella over 9 mm in diameter would significantly increase the risk of rejection. In this regard, the mushroom technique allows for the complete replacement of the exhausted endothelium of the old graft while preserving the patient’s peripheral endothelium, potentially minimizing the risk of endothelial rejection (Figure 11).

## 6. Conclusions

Our case series pilot results support the hypothesis that this procedure is effective in managing corneal graft ectasia combined with endothelial decompensation, as the patients showed significant and stable improvements in both corneal topographic parameters and corneal thickness. Visual outcomes were also satisfactory, with two patients obtaining a final BCVA lower than 0.4 LogMAR. The residual astigmatism was 3.0 D on average, and no episodes of endothelial rejection were reported.

This study has several limitations that should be acknowledged. The retrospective nature and, consequently, the absence of randomization or blinding may introduce selection bias, although criteria for inclusion and exclusion were established to isolate a targeted population of patients. Our case series of 15 patients is relatively small in size and lacks a control group, which limits the generalizability of the data even in the presence of statistically significant results. Finally, although the punch diameter of the lamellae remained fairly consistent among patients in the case series, with the posterior lamella ranging from 7.5 to 8.5 mm and the anterior lamella ranging from 9.0 to 9.5 mm, patients were not divided into subgroups based on graft diameter, which could have helped highlight differences in outcomes based on these parameters. These limitations underscore the need for further prospective studies with larger, more diverse populations and robust control groups to validate our observations.

In conclusion, this technique’s objective is to correct both stromal and endothelial issues in a single surgical procedure, maximizing outcomes in terms of visual acuity and post-operative astigmatism while simultaneously minimizing the risks of endothelial rejection and graft failure.

## Figures and Tables

**Table 1:** Baseline characteristics of patients in the case series. Numerical values are expressed in terms of the average ± StDev. * Preoperative EEC was not measurable due to the poor condition of the endothelium and edema.

AGE (years)	58 ± 9
GENDER	14 M; 1 F
EYE	7 OD; 8 OS
YEARS FROM PRIMARY PK	28.5 ± 13.3
BCVA (LogMAR)	1.1 ± 0.4
CCT (µm)	629 ± 39
ECD (cells/mm^2^)	not measurable *
KMIN (D)	46.8 ± 4.9
KMAX (D)	56.5 ± 5.1
AVK (D)	51.1 ± 4.5

**Table 2:** Post-operative results at 6 and 12 months. *p* values are calculated between the value at each time point and preoperatively. Numerical values are expressed as the average ± StDev. *p* values for ECD are not available due to a lack of accurate preoperative measurements.

Parameter Measured	6 Months		12 Months	
BCVA (LogMAR)	0.5 ± 0.3	*p* < 0.0001	0.3 ± 0.2	*p* < 0.0001
CCT (µm)	528 ± 46	*p* < 0.0001	532 ± 45	*p* = 0.0001
ECD (cells/mm^2^)	2181 ± 190		1926 ± 199	
KMIN (D)	41.7 ± 1.8	*p* < 0.001	42.0 ± 1.2	*p* = 0.001
KMAX (D)	45.3 ± 1.5	*p* < 0.0001	45.1 ± 1.2	*p* < 0.0001
AVK (D)	43.5 ± 1.4	*p* < 0.0001	43.5 ± 1.1	*p* < 0.0001

## Data Availability

The data is confidential due to the policies of the ethics committee.

## Supplementary Materials

The following supporting information can be downloaded at https://www.mdpi.com/article/10.3390/jcm14020343/s1. Video S1: Mushroom PK on post-PK ectasia: full procedure. Please note, microkeratome-assisted splitting of the donor cornea is performed without a microscope; therefore, it was not recorded.

## Abbreviations

PK, penetrating keratoplasty; DALK, deep anterior lamellar keratoplasty; EK, endothelial keratoplasty; AS-OCT, anterior segment optical coherence tomography; FSL, femtosecond-laser; BCVA, best corrected visual acuity; LEF, late endothelial failure; CCT, central corneal thickness; ECD, endothelial cell density.
